# Comment on “Flavoring Chemicals in E-Cigarettes: Diacetyl, 2,3-Pentanedione, and Acetoin in a Sample of 51 Products, Including Fruit-, Candy-, and Cocktail-Flavored E-Cigarettes”

**DOI:** 10.1289/ehp.1611350

**Published:** 2016-06-01

**Authors:** Jennifer S. Pierce, Anders Abelmann, Brent L. Finley

**Affiliations:** 1 Cardno ChemRisk, Chicago, Illinois, USA; 2 Cardno ChemRisk, Brooklyn, New York, USA

We read with interest the article by Allen et al. in which the authors reported the presence of diacetyl and 2,3-pentanedione in e-cigarette vapors and concluded that “[d]ue to the associations between diacetyl, bronchiolitis obliterans and other severe respiratory diseases observed in workers, urgent action is recommended to further evaluate this potentially widespread exposure.” As part of their analysis, it was suggested that the occupational exposure limits (OELs) that have been proposed for these compounds may not be health protective for most e-cigarette users.

We support efforts to characterize the composition of e-cigarette vapors and believe that such research is necessary to fully assess the potential health risks, if any, associated with use of these products. Over the past five years, we have published the results of several studies in which diacetyl and 2,3-pentanedione levels were measured in various consumer products (Gaffney et al. 2015; Pierce et al. 2014; Pierce et al. 2015). As described briefly below, we believe our findings are directly relevant to many of the issues raised by Allen et al.

First, it is important to understand that hundreds of consumer products (e.g., tea, coffee, citrus juices, butter) contain naturally occurring diacetyl and 2,3-pentanedione (Bartowsky and Henschke 2004; NTP 2007; Ganeko et al. 2008), and several studies have shown that airborne diketones associated with these products are easily detectable (Grosch and Mayer 2000; Mayer and Grosch 2001; Ott et al. 1999; Xanthopoulos et al. 1994). Hence, the mere presence of these compounds in a particular product is not indicative of a risk of lung disease, and we therefore agree with Allen et al. that estimates of potential consumer exposures are critical in evaluating the safety of a product.

Second, our work has shown that the naturally occurring diketone concentrations emitted from common consumer products can be far higher than the OELs that have been proposed by the National Institute for Occupational Safety and Health (NIOSH) or recommended by the American Conference of Governmental Industrial Hygienists (ACGIH). For example, Gaffney et al. (2015) and Pierce et al. (2015) found that grinding, brewing, and consuming unflavored coffee was associated with airborne diacetyl concentrations that were several times higher than the NIOSH and ACGIH short-term (0.025 and 0.020 ppm, respectively) and 8-hour (0.005 and 0.010 ppm, respectively) OELs for diacetyl. Unless one assumes that unflavored coffee beans pose a serious risk of “popcorn lung,” a rare and oftentimes lethal disease, then one should agree that exposures to airborne diketone levels above the NIOSH and ACGIH OELs are not necessarily indicative of respiratory risk.

Similarly, we measured concentrations of naturally occurring diacetyl and 2,3-pentanedione in mainstream cigarette smoke at levels (200–400 ppm and 30–50 ppm, respectively) that are hundreds of thousands of times higher than the NIOSH and ACGIH OELs, yet cigarette smoking is not associated with “popcorn lung” (Pierce et al. 2014). Also, as others have noted, diketone exposures from traditional cigarettes are higher than those associated with e-cigarette use (Farsalinos et al. 2015), hence switching from tobacco to e-cigarettes may result in reduced diketone exposure. This is a critical issue that was not mentioned by Allen et al. Indeed, based on the diketone levels measured in the vapors of the e-cigarette liquids evaluated by Allen et al. (median = 6.0 µg/e-cigarette for diacetyl and 1.6 µg/e-cigarette for 2,3-pentanedione), e-cigarette users likely experience inhaled diketone doses that are far below those associated with the NIOSH draft OELs (176 µg/day for diacetyl and 381 µg/day for 2,3-pentanedione, which correspond to the OELs of 0.005 ppm and 0.0093 ppm, respectively, assuming a breathing volume of 10 m^3^/workday) (EPA 2009).

We suggest that future research on this topic should include a re-evaluation of the proposed NIOSH OELs, in part because these values suggest that natural aromas from common foods pose a severe respiratory hazard. Elsewhere we have described what we believe to be shortcomings associated with these OEL values (Pierce et al. 2015). Regarding e-cigarettes specifically, we believe the following areas need to be addressed: *1*) the effects of vaping topography and device parameters on e-cigarette emissions, and *2*) the consumption patterns of users of e-cigarettes, such that true exposure to diketones and the other constituents of e-cigarette vapors can be evaluated. Ironically, suggesting that diketone levels in e-cigarettes are potentially dangerous could actually lead to higher diketone exposures in the smoking population if smokers decide not to switch to e-cigarettes due to as yet unfounded health concerns.

Finally, the question of whether the weight of evidence truly supports an increased risk of diketone-related “popcorn lung” or any other serious respiratory disease in flavorings-exposed workers is still open to debate (e.g., Clark and Winter 2015). As OSHA’s website currently states, “the causal relationship between diacetyl exposure and development of bronchiolitis obliterans has not been firmly established” (OSHA 2016).
